# Enhancing prognostic power in multiple myeloma using a plasma cell signature derived from single-cell RNA sequencing

**DOI:** 10.1038/s41408-024-01024-8

**Published:** 2024-03-06

**Authors:** Jian-rong Li, Shahram Arsang-Jang, Yan Cheng, Fumou Sun, Anita D’Souza, Binod Dhakal, Parameswaran Hari, Quillan Huang, Paul Auer, Yong Li, Raul Urrutia, Fenghuang Zhan, John D. Shaughnessy, Siegfried Janz, Jing Dong, Chao Cheng

**Affiliations:** 1 Department of Medicine, Baylor College of Medicine, Houston, TX USA; 2 Institute for Clinical and Translational Research, Baylor College of Medicine, Houston, TX USA; 3 Division of Hematology Oncology, Department of Medicine, Medical College of Wisconsin, Milwaukee, WI USA; 4 Myeloma Center, Winthrop P. Rockefeller Cancer Institute, Department of Internal Medicine, University of Arkansas for Medical Sciences, Little Rock, AR USA; 5 Department of Hematology/Oncology, Baylor College of Medicine, Houston, TX USA; 6 Division of Biostatistics, Institute for Health & Equity, and Cancer Center, Medical College of Wisconsin, Milwaukee, WI USA; 7 Linda T. and John A. Mellowes Center for Genomic Sciences and Precision Medicine, Medical College of Wisconsin, Milwaukee, WI USA; 8 Medical College of Wisconsin Cancer Center, Milwaukee, WI USA; 9 Dan L Duncan Comprehensive Cancer Center, Baylor College of Medicine, Houston, TX USA

**Keywords:** Risk factors, Cancer genetics

## Abstract

Multiple myeloma (MM) is a heterogenous plasma cell malignancy, for which the established prognostic models exhibit limitations in capturing the full spectrum of outcome variability. Leveraging single-cell RNA-sequencing data, we developed a novel plasma cell gene signature. We evaluated and validated the associations of the resulting plasma cell malignancy (PBM) score with disease state, progression and clinical outcomes using data from five independent myeloma studies consisting of 2115 samples (1978 MM, 65 monoclonal gammopathy of undetermined significance, 35 smoldering MM, and 37 healthy controls). Overall, a higher PBM score was significantly associated with a more advanced stage within the spectrum of plasma cell dyscrasias (all *p* < 0.05) and a shorter overall survival in MM (hazard ratio, HR = 1.72; *p* < 0.001). Notably, the prognostic effect of the PBM score was independent of the International Staging System (ISS) and Revised ISS (R-ISS). The downstream analysis further linked higher PBM scores with the presence of cytogenetic abnormalities, *TP53* mutations, and compositional changes in the myeloma tumor immune microenvironment. Our integrated analyses suggest the PBM score may provide an opportunity for refining risk stratification and guide decisions on therapeutic approaches to MM.

## Introduction

Multiple myeloma (MM) is the second most common hematological malignancy in the US [1]. It is characterized by the proliferation of clonal plasma cells in the bone marrow and represents the final stage in a continuum of plasma cell dyscrasias, arising from the premalignant conditions monoclonal gammopathy of undetermined significance (MGUS) and smoldering MM (SMM) [2]. The complex interplay between immune dysfunction and myeloma development and progression has garnered substantial attention, highlighting the potential for immune-based therapies. However, the heterogeneity of treatment response remains a major challenge, necessitating the identification of robust prognostic factors. In this context, genetic variations, including specific chromosomal abnormalities, have been linked to distinct clinical outcomes in MM [1, 3]. Established prognostic models such as the International Staging System (ISS) and the Revised ISS (R-ISS), incorporating genetic features alongside clinical parameters such as serum β2-microglobulin (B2M), albumin, and lactate dehydrogenase (LDH), have enabled risk stratification and informed treatment decisions [4, 5]. Microarray based gene expression models have also been developed to facilitate stratification for MM [6–10]. Nonetheless, these models exhibit limitations in capturing the full spectrum of outcome variability, prompting the exploration of novel prognostic and risk stratification approaches that leverage cutting-edge molecular profiling technologies.

In this study, we capitalize on the power of single-cell RNA sequencing (scRNA-seq) to dissect the intricate landscape of human immune cells and develop a novel gene signature specific to normal plasma cells [11]. By applying this signature to gene expression profiles of purified CD138+ cells, we calculate the plasma cell malignancy (PBM) score, which provides a precise and comprehensive assessment of the malignancy level in MM samples. To validate the clinical relevance of the PBM score, we extensively interrogated genomic and transcriptomic data from multiple large-scale MM cohorts, including the influential MMRF CoMMpass study, UAMS, MAQC-II, APEX phase 3 trial, and Mayo Clinic cohorts [12–16]. By investigating the associations between the PBM score and known MM driver genes, we shed light on molecular underpinnings of disease progression and uncover potential therapeutic targets.

## Methods

### Calculation of plasma cell malignancy score based on gene expression data

**Fig. 1:**
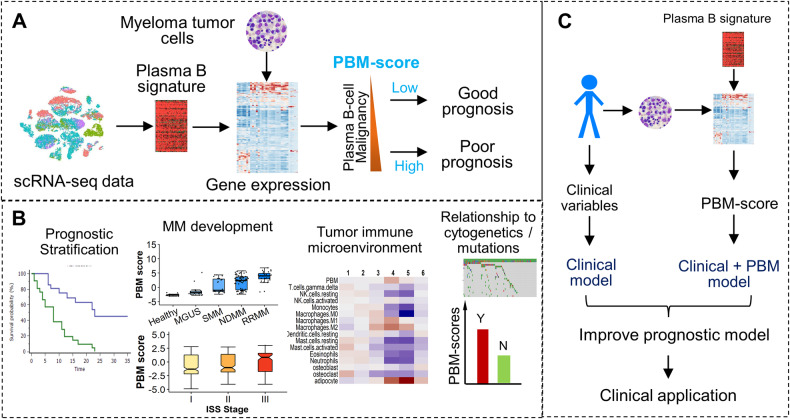
Overview of the study. **A** A plasma B-cell signature was defined to quantify the magnitude of malignancy of MM samples. The resulting plasma B malignancy (PBM) score is associated with the patient’s prognosis. **B** The PBM scores of CD138+ cells were found to be used for prognosis prediction, characterize the MM development and progression, and be correlated with the patient’s immune microenvironment and cytogenetic abnormalities. **C** The PB signature was incorporated into the prognostic model to improve prediction accuracy for better clinical application.

This study was approved by the Institutional Review Board at the Medical College of Wisconsin. The overall design of the current study was shown in Fig. 1. Based on an integrated scRNA-seq database-the PanglaoDB database, we defined marker genes for 16 different types of human immune cells, including three types of B cells (i.e., plasma B, naive B, and memory B cells) [11]. The marker genes for plasma B cells were used as a signature to calculate sample-specific plasma B-cell malignancy scores (PBM scores) based on the expression profiles of CD138+ cells collected from MM patients using microarray data. Specifically, a rank-based algorithm was applied to quantify the perturbed expression of plasma B-cell markers in CD138+ cells (see details in Supplementary Methods) [17]. A higher PBM score indicates more perturbed marker gene expression and therefore a higher level of malignancy of CD138+ cells. Similarly, sample-specific scores were also calculated for marker gene-based signatures of other immune cells.

### Patient cohort, transcriptomic and genomic data

Gene expression and/or genomic data from the CoMMpass, UAMS, MAQC-II, APEX phase 3 trial, and Mayo Clinic studies were used to investigate the clinical impacts of the PBM score on MM development and prognosis [12–16]. Details of each study and molecular data analysis are provided in the Supplementary Methods and Supplementary Table 1. Survival analysis was performed to investigate the associations between the PBM score and patient prognosis using RNA-seq data from the CoMMpass study and microarray data from the UAMS-I, MAQC-II, and APEX studies. The associations between PBM score and MM development and progressions were examined by using the microarray data from the Mayo Clinic and UAMS-II studies, which included gene expression profiles for samples from healthy controls, MGUS, SMM, newly diagnosed MM (NDMM), and relapsed/refractory MM (RRMM). In all datasets, the transcriptomic profiles represent gene expression in purified CD138+ cells. The UAMS-I dataset which also had transcriptomic profiles of CD138+ cells and matched bone marrow samples for 401 patients were used to investigate the associations between the PBM score and tumor immune microenvironment (TIME). The genomic data was used to examine the association between PBM scores and somatic mutations.

### Statistical methods

Comparison of PBM scores between groups was conducted using Wilcoxon’s rank-sum test. Survival was analyzed using the Kaplan–Meier method and log-rank tests. Hazard ratios (HRs) and 95% CIs were estimated using a Cox proportional hazard model. Concordance indices (C-index) were calculated to evaluate the performance of the PBM score in predicting MM survival. The Benjamin–Hochberg method was applied to calculate adjusted *p* values for multiple testing correction, e.g., in differential gene expression analysis. Significance was determined at a two-sided *α* level of 0.05. All statistical analyses were conducted in the R environment (v4.0.2). Details of the methods are provided in the Supplementary Methods.

## Results

### The development of plasma cell signature

**Fig. 2:**
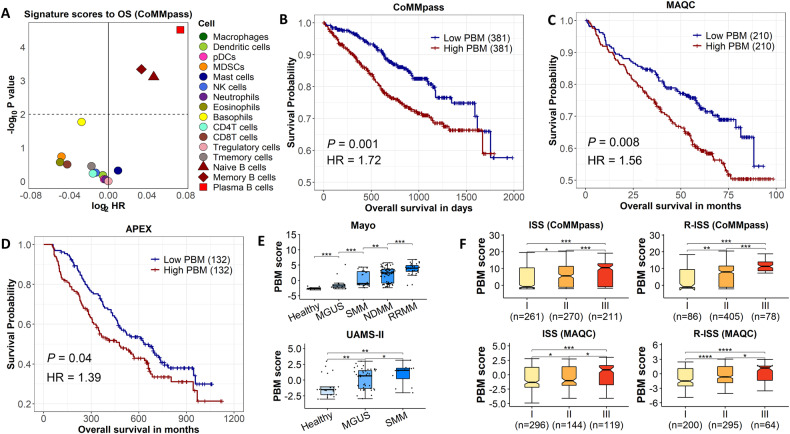
PBM score and MM development and prognosis. **A** The Cox regression *p* value and log2 hazard ratio (HR) of overall survival (OS) for MM patients to negative signature scores of 16 types of immune cells in the CoMMpass dataset. **B** KM-plot of OS to MM patients with high PBM scores versus patients with low PBM scores in the CoMMpass dataset. The *p* value is calculated by Cox regression, and the cutoff of high and low PBM groups is the median PBM score. The same is below. **C** KM-plot of OS to MM patients with high PBM scores versus patients with low PBM scores in the MAQC-II dataset. **D** KM-plot of OS to MM patients with high PBM scores versus patients with low PBM scores in the APEX phase 3 dataset. **E** PBM scores between healthy controls (*n* = 15), patients with monoclonal gammopathy of unknown significance (MGUS) (*n* = 21), smoldering multiple myeloma (SMM) (*n* = 23), newly diagnosed multiple myeloma (NDMM) (*n* = 75), and relapsed or refractory multiple myeloma (RRMM) (*n* = 28) in the Mayo Clinic dataset (upper panel). The lower panel is PBM scores between 22 healthy controls, 44 MGUS, and 12 SMM in the UAMS-II dataset. *p* value is calculated with Wilcoxon’s rank-sum test. **F** The PBM scores between stages I, II, and III of the ISS (left) and R-ISS (right) in the CoMMpass and MAQC-II studies. *p* value is calculated with Wilcoxon’s rank-sum test.

To examine whether the level of malignancy of CD138+ cells purified from MM samples can be quantified by examining the perturbed expression of normal plasma cell marker genes, we defined signatures for 16 different types of immune cells based on their marker genes (Supplementary Table 2). Each of these signatures was applied to the CD138+ cell expression profiles from the CoMMpass study to calculate a sample-specific score, a rank-based statistic that summarized the expression of signature genes (i.e., marker genes). In particular, the plasma cell signature score indicated the level of malignancy of patient MM samples (denoted as PBM score). The associations between each of the immune cell signature scores and MM survival were evaluated in 762 patients from the CoMMpass study. As shown in Fig. 2A, three immune cell scores were significantly associated with overall survival (OS) (*p* < 0.01), all of which were B cell derived scores. As expected, the score of plasma B cell (i.e., PBM score), exhibit the most significant association (*p* < 0.001). These results indicated that the PBM scores of CD138+ samples provided a quantitative measurement of their malignancy levels.

### Investigations on the impact of plasma cell malignancy provides prognostic insights and dynamic progression in MM

To investigate the influence of plasma cell malignancy in MM development and prognosis, we categorized the PBM score into “high” and “low” groups using the median as the threshold. A higher PBM score was identified to be significantly associated with a shorter OS (HR = 1.72, *p* < 0.01) (Fig. 2B). The impact of the PBM score on the OS remained significant after controlling for age, sex, race, and the ISS stage (HR = 1.50, *p* = 0.01, Supplementary Fig. 1A). The prognostic value of the PBM score in OS was further validated using independent datasets from the MAQC-II (423 MM) and APEX (264 MM) studies (Fig. 2C, D). In addition, we found that a higher PBM score was also associated with a worse event-free survival (EFS) in patients with MM (Supplementary Fig. 1B–D).

We then examined how PBM score changes during MM development and progression. Using data from the Mayo Clinic study, we calculated PBM scores for healthy controls (*n* = 15) and patients with MGUS (*n* = 21), SMM (*n* = 23), NDMM (*n* = 75), and RRMM (*n* = 28). A gradual increase in the PBM score was observed during disease progression, with a higher PBM score present in the patient groups compared with healthy controls, and in patients with more advanced disease compared with those with less advanced disease (*p* for trend <0.001, Fig. 2E). These findings were further validated using an independent dataset from the UAMS-II study, consisting of 22 healthy controls, 44 MGUS, and 12 SMM patients (Fig. 2E). A gradual and significant increase in the PBM score was also observed as MM advanced from stage I to stage III of both ISS and R-ISS (Fig. 2F). Collectively, these results indicate that the PBM score can serve as a quantifiable measure of the severity of the plasma cell malignancy, not only in patients with MM but also in patients with premalignant conditions.

### Deriving plasma cell malignancy and MM molecular characteristics from cytogenetic abnormalities, molecular subgroups, and pathway enrichment

**Fig. 3:**
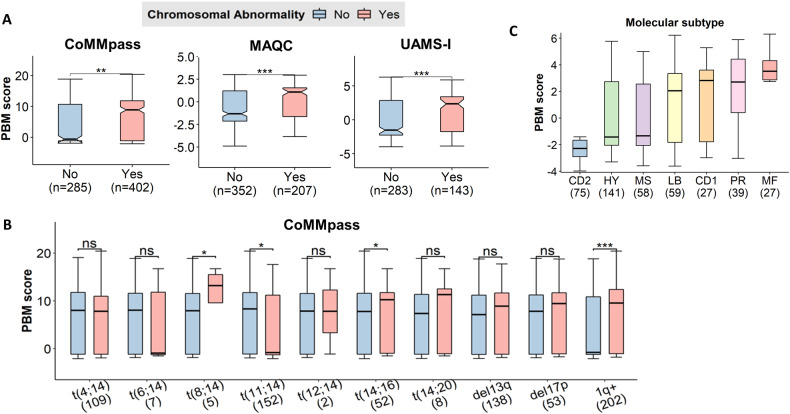
The PBM scores are associated with the intrinsic molecular characteristics of MM. **A** Patients with cytogenetic abnormalities had higher PBM scores than those without in the CoMMpass, MAQC-II, and UAMS-I datasets. **B** Among the ten cytogenetic abnormalities, the difference in PBM scores between patients with this cytogenetic abnormality and those without in the CoMMpass dataset. **C** PBM scores of patients that were classified into the UAMS molecular subtype groups (CD1, CD2, HY, LB, MF, MS, and PR) in the UAMS-I dataset.

To explore the biological relevance of the PBM score in MM, we first investigated its relationship with cytogenetic abnormalities using data from the CoMMpass study. Overall, MM patients with detectable cytogenetic abnormalities had a significantly higher PBM score than patients who had no cytogenetic abnormalities (*p* < 0.001, Fig. 3A). These results were verified in the MAQC-II and UAMS-I datasets (Fig. 3A). Analysis for individual cytogenetic abnormality in the CoMMpass study observed a significantly higher PBM score among MM patients with 1q gains (1q+), *t*(8;14), *t*(11;14), and *t*(14;16) compared with patients without these abnormalities (Fig. 3B).

Next, we tested associations between the PBM score and the seven MM molecular subgroups (i.e., CD1, CD2, HY, LB, PR, MF, and MS) that were defined based on gene expression data from the UAMS-I study. The CD2 subgroup showed the lowest PBM scores, while the MF subgroup showed the highest PBM scores (Fig. 3C). Interestingly, the CD1 and CD2 subgroups have similar expression pattern [18], however, they vary significantly in their PBM scores (*p* < 0.001). We combined the subgroups with favorable survival outcomes (CD-1, CD-2, HY, and LB) and adverse survival outcomes (PR, MF, and MS), respectively, and found that compared to patients with adverse molecular subtypes, patients with favorable subtypes had a significant lower PBM (*p* < 0.001).

We further conducted pathway enrichment analysis using Gene Ontology (GO) for genes which expression in CD138+ cells were correlated with PBM scores (Supplementary Table 3). We found that positively correlated genes were enriched in tumor proliferation-related pathways, such as ribosome biogenesis and chromosome segregation, while negatively correlated genes were enriched in immune response or receptor signaling pathways (Supplementary Tables 4 and 5).

### Correlation between PBM score and myeloma driver mutations: insights from tumor mutation burden analysis

**Fig. 4:**
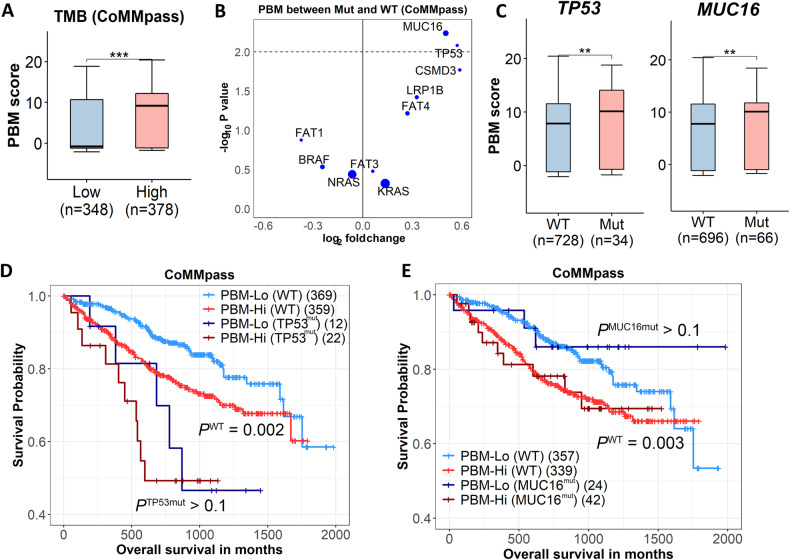
PBM score and MM driver mutations. **A** Patients with higher tumor mutation burden (TMB) have higher PBM scores than those with lower TMB. TMB cutoff: median. Wilcoxon’s rank-sum test. **B** The PBM scores of patients with a given nonsynonymous gene mutation in ten genes compare to the PBM scores of patients with wild-type of the gene. The size of each point indicates the sample size of the mutated gene. *p* value is generated using Wilcoxon’s rank sum test. **C** The PBM scores of patients with *TP53* or *MUC16* mutations compared to that of patients carrying wild-type. **D** KM-plot of OS for patients with high PBM scores versus those with low PBM scores stratified by *TP53* mutation status using the CoMMpass dataset. *p* values are calculated by Cox regression, and the cutoff of high and low PBM groups is the median PBM score of all patients. **E** KM-plot of OS for patients with high PBM scores versus those with low PBM scores stratified by *MUC16* mutation status using the CoMMpass dataset.

Overall, myeloma patients with a higher PBM score tend to have a larger number of non-synonymous somatic mutations (i.e., tumor mutation burden, TMB) than patients with a lower PBM score (*p* < 0.001, Fig. 4A), indicating that there might exist certain somatic mutations that drive the severity of plasma cell malignancy. To identify the potential driver mutations, we examined the top 10 most frequently mutated COSMIC Cancer genes with nonsynonymous mutations in at least 30 of the 762 MM patients in the CoMMpass study (Supplementary Table 6). We found that the PBM score was significantly increased in patients carrying *TP53* or *MUC16* mutations compared to those with wild-type alleles (Fig. 4B, C). Intriguingly, PBM scores could predict patients’ survival even among those who don’t carry *TP53* or *MUC16* mutations, with log-rank *p* values of 0.002 and 0.003 for *TP53* or *MUC16* wide-type carriers, respectively (Fig. 4D, E).

### Significant impact of plasma cell malignancy on tumor immune microenvironment

**Fig. 5:**
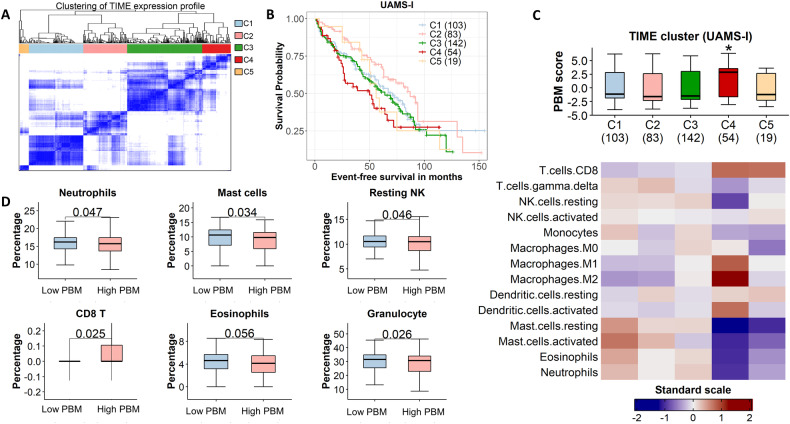
The TIME clusters have different PBM score in CD138+ cells shows that the malignancy of MM affects its TIME. **A** Clustering using TIME expression profiles derived 5 clusters in the UAMS-I dataset. **B** KM-plot of 5 clusters in the UAMS-I dataset. Cluster-4 (C4) has poor PFS than others, and C2 has the best prognosis; **C** The TIME expression cluster 2 has the lowest PBM scores (average PBM score = −0.19), and cluster 4, characterized by low infiltration of granulocytes and higher infiltration of M1/M2 macrophages in TIME, had significantly higher PBM scores (average PBM = 1.46) than other clusters. The *p* values of clusters 1, 2, 3, and 5 compared to cluster 4 are 0.009, 2e−04, 4e−05, 0.008, 0.029, respectively. Wilcoxon’s rank-sum test. The heatmap of TIME removed cells with an average of less than 1% per sample, as well as osteoblast, osteoclast, and adipocyte. The values for each cell are presented as a distribution normalized to a mean of 0 and a standard deviation of 1. **D** MM patients with high PBM scores in CD138+ cells in all samples of the UAMS-I dataset have lower neutrophils, mast cells, resting NK cells, and higher CD8 T cell infiltration in their TIME.

The UAMS-I study provides paired gene expression profiles for both tumor CD138+ and whole bone marrow (WBM) samples, enable us to simultaneously calculate the PBM score and infer immune infiltration in the TIME. To gain insights into the impact of plasma cell malignancy on the TIME, we performed clustering analysis to divide patients into 5 distinct clusters based on their immune gene expression profiles. Among them, cluster-2 (C2) shows the best survival (*p* = 0.016) while cluster-4 (C4) exhibits the worst survival (*p* = 0.048) (Fig. 5A, B). Consistently, C2 exhibits the lowest PBM score, while C4 presents the highest PBM score. C4 displayed a distinct TIME composition characterized by lower infiltration of NK resting cells and granulocytes (e.g., neutrophils, mast cells, and eosinophils) but higher infiltration of CD8 T-cells and M1/M2 macrophages (Fig. 5C). The PBM scores derived from CD138+ cells were correlated with immune cell infiltration levels. As shown in Fig. 5D, MM samples with higher PBM scores showed significantly lower granulocytes and NK resting cells but higher CD8+ T-cells in the TIME. These data suggest the mutual interactions between malignant plasma B cells and the TIME of MM, which is correlated with MM prognosis.

### Enhanced prognostic performance of PBM score in predicting MM survival

**Fig. 6:**
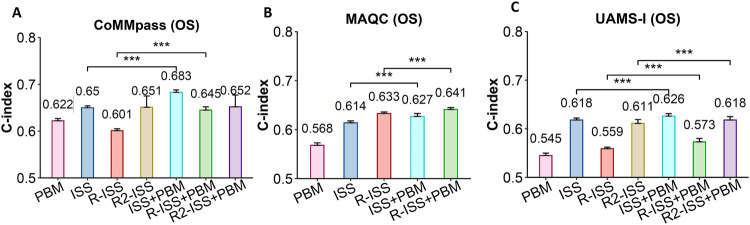
Adding PBM scores to the prognosis model can improve prediction accuracy. **A** The C-index (concordance index) of ISS, R-ISS or R2-ISS in predicting MM OS with and without the inclusion of the PBM score in the Cox models in the CoMMpass dataset. The *p* values were calculated by comparing the C-indexes calculated from 100 times 5-fold cross-validation Cox models using Wilcoxon’s rank-sum test. **B** The C-index of ISS or R-ISS in predicting MM OS with and without the inclusion of the PBM score in the Cox models in the MAQC-II dataset (note: MAQC-II dataset does not include cytogenetic information so R2-ISS cannot be calculated). **C** The C-index of ISS, R-ISS or R2-ISS in predicting MM OS with and without the inclusion of the PBM score in the Cox models in the UAMS-I dataset.

We evaluated the predictive performance of ISS, R-ISS, and R2-ISS in predicting MM survival with and without the inclusion of the PBM score in the models. Using the CoMMpass dataset, the model based on the PBM score alone had a c-index of 0.62 to predict the OS of MM. The clinical models based on ISS, R-ISS, and R2-ISS had a c-index of 0.65, 0.60, and 0.65, respectively. Incorporating the PBM score into the clinical models significantly improved the prognostic performance of the models, with the c-index increased to 0.68 (ISS + PBM) and 0.65 (R-ISS + PBM), respectively (both *p* < 0.001) (Fig. 6A). Similar results were observed when using the datasets from the MAQC-II and UAMS-I studies (Fig. 6B, C), as well as investigating the performance of the PBM score in predicting EFS (Supplementary Fig. 2). Of note, for the R2-ISS model, the c-index didn’t change much when adding the PBM score to the model in the CoMMpass study, while the c-index significantly increased from 0.61 to 0.62 in predicting OS and from 0.609 to 0.614 in predicting EFS in the UAMS-I study. We also compared the predictive performance of the PBM score with other prognostic markers, such as double hit (DH) and increased LDH, in predicting MM survival. As shown in Supplementary Fig. 3, both DH and increased LDH have a low to moderate performance to predict the OS of MM, with a c-index of 0.56 and 0.50 for DH and a c-index of 0.56 and 0.51 for increased LDH in the CoMMpass and UAMS-I studies, respectively. As expected, adding the PBM score into the models based on DH and increased LDH significantly improved the prognosis performance of the models, with the c-index increased to 0.60 and 0.54 for DH, and 0.62 and 0.54 for increased LDH in the CoMMpass and UAMS-I studies, respectively (*p* < 0.001, Supplementary Fig. 3A–C). Similar results were observed when investigating the performance of DH and increased LDH in predicting EFS (Supplementary Fig. 3D, E).

### Leveraging the PBM score to distinguish different stages of plasma cell dyscrasias

Because the PBM score was not only prognostic of MM, but also associated with the progression and malignant transformation of MM, we further evaluated the performance of PBM score in distinguishing different stages of plasma cell dyscrasias. Using the Mayo Clinic dataset, the PBM score had the AUC of 0.87, 0.99, 0.99, and 1.00 to distinguish healthy controls from patients with MGUS, SMM, NDMM and RRMM, and AUC of 0.78, 0.88 and 0.94 to differ MGUS from patients with SMM, NDMM and RRMM (Supplementary Table 7). When comparing to the previously reported prognostic signatures GEP70 and SKY92, the PBM score had a better risk stratification performance, especially for distinguishing patients with premalignant conditions (i.e., MGUS and SMM) (Supplementary Table 7).

**Fig. 7:**
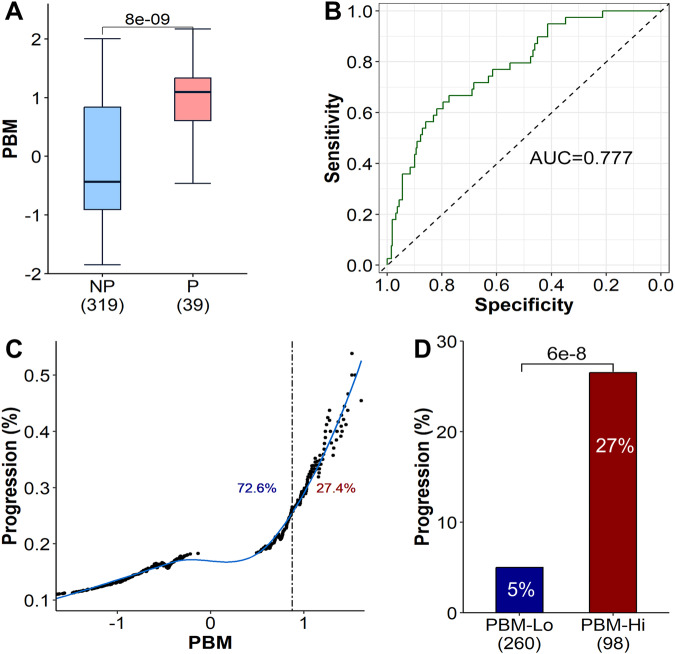
PBM scores as predictors of the progression from MGUS to MM. **A** Comparison of PBM scores between MGUS patients who progressed to MM (P) and those who did not progressed (NP) to MM. PBM scores are significantly higher in the “P” group compared to the “NP” group. *p* values were calculated using the Wilcoxon rank-sum test. **B** Receiver operating characteristic (ROC) curve for predicting progression to MM using PBM scores. **C** Proportion of MGUS patients with progression exceeding the critical PBM threshold, determined individually for each patient. The dashed line represents the optimal PBM cutoff of 0.875 as derived from the ROC curve, with percentages indicating the rate of patients categorized into low and high PBM groups based on this cutoff. **D** Proportions of MGUS patients who progressed to MM in high and low PBM groups. *p* values are calculated using Fisher’s exact test.

Utilizing the gene expression profiles from our recent study in 358 MGUS (319 did not progress to MM and 39 progressed to MM) with longitudinal data detailing their progression to MM (GSE235356) [19], we further investigated whether the PBM score could predict the progression of MGUS to MM. We found that MGUS patients who eventually progressed to MM had significantly higher PBM score compared to those not progressed (*p* < 0.001, Fig. 7A). The PBM score had the AUC of 0.777 to predict the progression of MGUS to MM (Fig. 7B), with an optimal cutoff value for PBM being 0.875 (specificity = 0.774, sensitivity = 0.667). As depicted in Fig. 7C, the proportion of MGUS patients who progressed to MM increased as the PBM score increased, particularly among those with PBM score exceeding 0.875. The rate of progression was over five times higher in the group with higher PBM score when compared to groups with lower PBM scores (*p* < 0.001, Fig. 7D).

## Discussion

This study makes a significant contribution to the field of precision oncology in MM by introducing the PBM score as a novel measurement to assess the deregulation of marker genes in normal plasma cells and quantify the magnitude of plasma cell malignancy in MM patients. We identified and validated the associations between an increased PBM score and a worse prognosis of MM. More importantly, the prognostic effects of the PBM score were independent of the currently available clinical scoring system (e.g., ISS/R-ISS), and integrating the PBM score could improve the performance of ISS/R-ISS in predicting MM prognosis. One particularly intriguing finding of this study is the association between an increased PBM score and the progression and malignant transformation of MM. This evidence not only strengthens the prognostic utility of the PBM score but also highlights its potential in the classification and risk stratification of both MM patients and individuals with premalignant conditions. By capturing specific genomic characteristics with prognostic or predictive value, the PBM score offers a promising avenue for precision medicine approaches in MM.

Gene expression profiling (GEP) in MM is an important step in deciphering the biological and clinical heterogeneity of the disease. Several GEP signatures, such as SKY92-, IFM15-, and UAMS GEP70-gene signatures have been developed to identify MM with poor outcomes [7, 20–22]. However, most of these studies simply correlated GEP with myeloma survival in the corresponding cohort, thus only very few or no genes were overlapped between these signatures, raising the concerns of reproducibility of these signatures [23, 24]. In the current study, we applied a novel signature enrichment-based method to instead utilize the entire transcriptome of plasma B-cell and calculated a PBM score to infer perturbed gene expression in myeloma cells. The prognostic effect of the PBM score was discovered in the RNA-seq dataset and validated in independent microarray datasets, underscoring the reliability and robustness of our findings. Unlike the previously reported GEP signatures that focused on survival outcomes, the PBM score could also distinguish between healthy controls and patients with MGUS, SMM, NDMM and RDMM, and exhibit better performance than the GEP70 and EMC92 signatures in distinguishing between healthy controls, premalignant conditions, and MM patients.

The downstream analysis of the PBM score has yielded several noteworthy insights into its biological relevance. For example, we observed significant associations between a higher PBM score and the presence of CA, especially those as high-risk CA (e.g., 1q gain and *t*(14;16)) that have been used in the R-ISS or the recently proposed R2-ISS and cytogenetic Prognostic Index [5, 25, 26]. The wide variations in MM clinical outcomes are largely driven by CA [1, 27]. Our findings suggest that the PBM score may capture certain genomic characteristics with prognostic or predictive information in MM. In addition, several recurrent mutations affecting MAPK (e.g., *KRAS*, *NRAS*, and *BRAF*), NF-κB (e.g., *TRAF3* and *CYLD*), and DNA-repair (e.g., *TP53* and *ATM*) pathways have been identified as secondary driver events in myelogenesis [3]. Despite most of these driver mutations are not prognostic for MM, we found that *TP53* mutation was associated with worse survival and a higher PBM score in MM. However, only less than 10% of the patients carry *TP53* mutations, largely limiting the clinical application. The prognostic stratification of the PBM score in patients carrying wild-type *TP53* provided a new avenue for the utilization of *TP53* mutation in clinical settings. *MUC16* mutation was also found to be associated with a higher PBM score. *MUC16* encodes cancer antigen 125 (CA-125) and is frequently mutated in solid tumors [28]. To date only one study investigated *MUC16* mutation in MM and reported a mutation rate of 6% [29]. In the CoMMpass study, the mutation rate of *MUC16* is a bit higher (8%), though no association was observed between *MUC16* mutation and MM survival. Future studies are needed to investigate the role of *MUC16* in myeloma biology.

Furthermore, the study highlights the intricate interplay between myeloma biology and innate and adaptive immune system dysfunction. Alterations of the normal bone marrow TIME leading to tumor escape from immunosurveillance are critical determinants of myelomagenesis and immunotherapy efficacy [30]. In the current analysis, we found that higher PBM scores are related to lower infiltration of the granulocytes and resting NK cells and higher infiltration of CD8 T-cells in TIME. This result is consistent with the report that the adverse outcomes of MM patients was correlated with elevated CD8+ T cell and reduced granulocytic cell proportions in the TIME [13]. Functional granulocytes can suppress T cell responses in a similar way as myeloid-derived suppressor cells (MDSCs) and are important for the growth of malignant plasma cells in MM, while activation of resting NK cells could induce natural cytotoxicity and cytokine secretion [31, 32]. Cytotoxic CD8 T-cells are dominant effectors of host control of the myeloma clone [33]. These findings suggest that the associations of PBM score in MM development and prognosis may reflect the immunological landscape of MM, with implications for immunotherapeutic interventions.

Here we show that the plasma cell signature characterizes the degree of CD138+ cell malignancy and is predictive of MM prognosis. However, the signature is not specifically defined for prognostic prediction. The whole transcriptomic profile for a MM sample is needed to calculate its PBM score. In addition, although adding PBM score improved the prognostic performance of the ISS/R-ISS model, the absolute magnitude of improvement may not be sufficient to justify its clinical use. To overcome these limitations, we may further optimize this signature by selecting a subset of prognostic genes and calculating prognostic scores solely based on their expression levels. Furthermore, although an increased PBM score was associated with the progression and malignant transformation of MM, we are not able to compare the performance of the PBM score with other known risk factors of progression, such as the FLC ratio, plasma cell percentage in the bone marrow, and serum M-protein level, due to data availability. In the UAMS-II study, 26 MGUS and 11 SMM patients have the data of FLC ratio, plasma cell percentage in the bone marrow, serum M-protein level, and 20/2/20 score available. We, therefore, compared the distributions of the PBM score and these variables between MGUS and SMM and found that only the PBM score and plasma cell percentage in the bone marrow were significantly different between MGUS and SMM (data not shown), indicating the predictive value of PBM score in differing MGUS and SMM.

In conclusion, this study represents an advance in the field of precision medicine for MM by introducing and validating the PBM score as a predictive tool. The independent prognostic information provided by the PBM score, beyond the established clinical scoring systems, provides an opportunity for refining risk stratification and guide decisions on therapeutic approaches to MM.

### Supplementary information

Supplementary Methods and Figures

Supplementary Table 1

Supplementary Table 2

Supplementary Table 3

Supplementary Table 4

Supplementary Table 5

Supplementary Table 6

Supplementary Table 7

## Data Availability

Microarray data are available at GEO under accession numbers GSE136400, GSE5900, GSE24080, GSE9782, and GSE6477. Genomic data are available at the dbGaP under the study accession phs000748.v7.p4 and the NCI’s Genomic Data Commons (GDC) upon request.
