# Supplementary material for: Enhancing prognostic power in multiple myeloma using a plasma cell signature derived from single-cell RNA sequencing
Source: Blood Cancer J. 2024 Mar 6;14(1):38. doi: 10.1038/s41408-024-01024-8 (PMC10915134; doi:10.1038/s41408-024-01024-8)

Supplementary Methods

Dataset Descriptions

Gene-expression and/or genomic data from the Multiple Myeloma Research Foundation (MMRF)-Clinical Outcomes in Multiple Myeloma to Personal Assessment of Genetic Profiles study (CoMMpass), the University of Arkansas for Medical Sciences (UAMS), the MicroArray Quality Control (MAQC)-II, the Assessment of Proteasome Inhibition for Extending Remissions (APEX) phase 3 trial and Mayo Clinic studies were used for the current analysis (Supplementary Table 1).[1-5] RNA-seq and whole-exome sequencing (WES) data from the CoMMpass study was obtained from the NCI Genomic Data Commons (GDC) Data Portal.[6] Gene-expression data of the UAMS, MAQC-II, APEX, and Mayo Clinic studies were obtained from the Gene Expression Omnibus (GEO) database.[7] To decrease the variability and make the expression profiles conform more closely to the normal distribution for the subsequent estimation of signature scores, the expression data from the CoMMpass, UAMS, MAQC-II, APEX, and Mayo Clinic studies were log2-transformed after adding a pseudo-count of 1.

The MMRF CoMMpass Study: The CoMMpass study is a large-scale, longitudinal study initiated in 2011 to better understand the molecular and genetic components of MM. Deidentified RNA-seq read counts and WES mutation data of purified tumor CD138+ cells from the bone marrow (BM) samples were obtained from the NCI GDC Data Portal.[6] The RNA-seq read counts were converted to FPKM (Fragments Per Kilobase of transcript per Million mapped reads) for normalization. The expression profiles of 32,594 genes in 859 samples were generated. Among them, the expression profiles of 762 samples with corresponding clinical information were selected for the current study and used as the training set in the survival analysis.

The UAMS Study: Two gene expression datasets of the UAMS study were used in our analysis. UAMS-I (GEO accession number: GSE136400) included 426 patients with newly diagnosed MM (NDMM) enrolled between 2004 and 2019.[2] Gene expression profiles were generated using Affymetrix Human Genome U133 Plus 2.0 Array in 867 whole bone marrow (WBM) samples, for which 401 had matched purified tumor CD138+ cells.[2] UAMS-I study was used as a validation set for the survival analysis. UAMS-II (GEO accession number: GSE5900) profiled gene expression of CD138-selected cells from the BM of 22 healthy controls, 44 MGUS, and 12 SMM patients using Affymetrix Human Genome U133 Plus 2.0 Array.[8] UAMS-II data was used for the analysis of MM development.

The MAQC-II Study: The MAQC-II study consists of 36 independent teams analyzing six microarray datasets to generate predictive models for lung or liver toxicity in rodents, breast cancer, MM, or neuroblastoma in humans.[3] The MM dataset (GEO accession number: GSE24080) including 559 NDMM was contributed by the Myeloma Institute for Research and Therapy at the UAMS. Gene expression profiling of highly purified bone marrow plasma cells was performed with Affymetrix Human Genome U133 Plus 2.0 Array and used as a validation set in the survival analysis. We noticed that there were 136 samples overlapped between the UAMS-I and MAQC-II datasets and have removed the expression data of the 136 samples from the analysis of MAQC-II study.

The APEX Study: This dataset included patients with relapsed myeloma enrolled in phase 2 and phase 3 clinical trials of bortezomib.[4] Pretreatment tumor CD138+ cells from the BM were

profiled with Affymetrix Human Genome U133A/B Array. Gene-expression data (GEO accession number: GSE9782) were generated in 264 patients from the APEX phase 3 trial (n=213), the SUMMIT phase 2 trial (n=44) and CREST phase 2 trial (n=7).[4,9-11] For simplicity, we used "APEX" to represent this study in our analysis. The APEX dataset was used as a validation set in the survival analysis.

The Mayo Clinic Study: The Mayo Clinic study included 15 healthy controls, 21 MGUS, 23 SMM, 75 NDMM, and 28 relapsed/refractory MM (RRMM) patients.[12] Gene expression (GEO accession number: GSE6477) was performed on CD138-selected BM cells using the Affymetrix Human Genome U133A chip. The Mayo Clinic dataset was used in the analysis of MM development.

Development of the Plasma B-cell Signature

To define the plasma cell signature, we referred to the immune cell specific marker genes curated by the PanglaoDB database.[13] The database integrated a large number of single-cell RNA-seq datasets from previous publications and defined high-resolution human immune cell clusters.[13] These clusters were corresponding to 16 different types of the human immune cell types, including naive B-cell, memory B-cell, and plasma B-cell. The data do not include malignant B cells, e.g., CD138+ cells from MM samples. The database manually curated a list of candidate marker genes for each immune cell type based on 4,598 previous publications. Following that, a ubiquitousness index (UI) and a specificity index (SI) were calculated to quantify the specificity of these candidate markers. Based on the provided information, we selected 84 genes specifically expressed in normal plasma B-cells to form the plasma B-cell (PB) signature (Supplementary

Table 2). As controls, we also defined signatures for naive B-cell, memory B-cell, and 13 other immune cell types (macrophage, dendritic cell, plasmacytoid dendritic cell, myeloid-derived suppressor cell, mast cell, natural killer cell, neutrophil, eosinophil, basophil, CD4+ T-cell, CD8+ T-cell, regulatory T-cell, memory T-cell) in a similar way.

Calculation of the Plasma B-Cell Malignancy Score

Given a MM gene expression dataset, we used the plasma B-cell signature to calculate a patient-specific score named plasma B-cell malignancy score (PBM score). PBM score quantifies the level of malignancy of CD138+ cells in MM tumors, with a greater score indicating a higher level of malignancy. To calculate the PBM score, a rank-based statical framework called BASE (Binding Associated with Sorted Expression)[14] was applied to gauge the perturbed expression of PB signature genes. Briefly, the analysis includes the following steps. First, in a given MM gene expression dataset, the expression of each gene was log-transformed and normalized by subtracting its median expression across all samples. Second, the expression of all genes in each sample was sorted in the decreasing order. Third, the expression values of all signature genes were summarized considering their positions in the ranked gene list, resulting in a raw enrichment score for each sample. Finally, the raw score was normalized against the null distribution obtained from permutated data to obtain the PBM score. Of note, the BASE algorithm was originally designed for weighted gene signatures.[15,16] Here we applied it to the unweighted gene sets. In this way, the BASE algorithm give rise to similar results with those from the single-sample GSEA analysis.[17]

Gene Ontology Biological Process Analysis

The Gene ontology (GO) biological process (BP) enrichment analysis was performed to explore the biological functions of genes correlated with PBM scores. Using the CoMMpass data, we identified genes that were correlated with the PBM scores in their expression values across all MM samples. In total, 234 positively correlated genes (Spearman correlation coefficient $r >$ 0.25, $P < 0.001$) and 1,075 negatively correlated genes ($r < -0.25$ and $P < 0.001$) were selected (Supplementary Table 3). GO enrichment analysis was implemented using the R package "*clusterProfiler*". We focused on biological process GO terms in our analysis.[18]

Tumor Microenvironment Immune Cell Inference and Clustering Analysis

The UAMS-I dataset has gene expression profiles for paired WBM and tumor CD138+ cell samples of 401 patients. By referring to the method described by Danziger et al[2], we inferred the infiltration levels of major immune cell types based on deconvolution analysis. Specifically, the digital cell quantifier (DCQ) algorithm, implemented by an R package "*ADAPTS*", was used to deconvolute WBM expression profiles.[19,20] A signature matrix named MGSM27 was used as the reference for deconvolution, which included the 22 leukocyte signature matrix (LM22), 4 bone marrow cell types (adipocyte, osteoclast, plasma memory cell, and osteoblast), and the malignant plasma B cell.[20,21]

After deconvolution analysis, we obtained the proportion of the above-list 27 cell types in each WBM sample. Then we combined these results with CD138+ cell expression data to calculate TIME-specific (CD138- cells) gene expression levels of all genes. Specifically, the proportion of the CD138+ cells ($Prop^{CD138+}$) in each WBM sample was estimated as the sum of the

plasma.cells, B.cells.memory, MM.plasma.cells, and PlasmaMemory.[2] The expression profiles of the CD138- cells (e^{CD138-}) in the WBM of each patient were then estimated as the following:

$$e^{CD138-} = (e^{WBM} - e^{CD138+} \times Prop^{CD138+})/(1 - Prop^{CD138+})$$

Based on the estimated CD138- expression profiles, we performed unsupervised clustering analysis by using the top 5% most expressed genes (n=805 genes). Specifically, the R package "*ConsensusClusterPlus*" was applied to implement this analysis, leading to 5 MM clusters as reported in the original study.[2,22]

The Selection of Myeloma Driver Mutations

To investigate the relationship between PBM score and myeloma driver mutations, 33 genes with nonsynonymous somatic mutations in at least 30 samples in the CoMMpass dataset were listed as candidates (Supplementary Table 6). Ten out of the 33 genes that were also included in the COSMIC Cancer Gene Census (CGC)[23] were selected for the subsequent analysis, including *KRAS*, *NRAS*, *MUC16*, *BRAF*, *FAT4*, *LRP1B*, *FAT3*, *CSMD3*, *FAT1*, and *TP53*.

Integrative Models for Predicting Prognosis

To examine the additional prognostic value contributed by the PBM score, we constructed prognostic models that include clinical variables with and without PBM scores. The performance of these models was evaluated and compared based on a 5-fold cross-validation. Specifically, the samples in a given dataset were randomly divided into 5 subgroups. Each time, 4 subgroups were selected and merged into the training data, and the other subgroup was used as the test data. The training data were used to fit a multivariate Cox regression model, which was subsequently used to predict the prognostic risk of samples in the test data. This procedure was rotated 5 times until

the prognostic risk of all samples was predicted exactly once. The predicted prognostic scores were compared with known survival information to evaluate the performance of the models. The performance of all models was evaluated 100 times, each from different subgroup randomization. The R package "*rms*" was used for model construction and evaluation. Specifically, the "*cph*" function was used to construct the univariate and multivariate Cox regression models; the "validate" function was used to perform 5-fold cross-validation for each model; and the "*survest"* function was used to calculate the prognostic risk (*i.e.,* survival probabilities) of samples in the testing dataset. The "*rcorr.cens*" function of the R package "*Hmisc*" was used to calculate the Concordance index (C-index) of prognostic models.

Statistical Analysis

The R package "*survival*" was implemented to perform the survival analysis. Univariate and multivariate Cox proportional hazard models were constructed to estimate the association between PBM score and patient survival by calculating hazard ratios (HRs) and 95% confidence intervals (CIs). By using the median of PBM scores as the threshold, MM patients were stratified into two groups with "high" and "low" PBM scores, respectively. Kaplan-Meier method was used to plot their survival curves and log-rank test was used to compare their survival difference. The difference between two samples groups were conducted using the Wilcoxon rank-sum test, and further confirmed by the Student's t-test for values with a normal distribution. The Benjamini-Hochberg method was used to correct multiple testing, e.g., in differential gene expression analysis. Receiver operating characteristic (ROC) analysis using PBM to predict the progression of MGUS was performed using the "*roc*" function of the R package "*pROC*". The cutoff value of the optimal PBM and the calculation of the area under the ROC curve (AUC) are

conducted using the same function. All statistical analyses were conducted in the R environment (v4.0.2).

REFERENCES

1. Keats JJ, Craig DW, Liang W, Venkata Y, Kurdoglu A, Aldrich J, et al. Interim Analysis Of The Mmrf Commpass Trial, a Longitudinal Study In Multiple Myeloma Relating Clinical Outcomes To Genomic and Immunophenotypic Profiles. *Blood*. 2013;122(21):532-532.
2. Danziger SA, McConnell M, Gockley J, Young MH, Rosenthal A, Schmitz F, et al. Bone marrow microenvironments that contribute to patient outcomes in newly diagnosed multiple myeloma: A cohort study of patients in the Total Therapy clinical trials. *PLoS Med*. 2020;17(11):e1003323.
3. Shi L, Campbell G, Jones WD, Campagne F, Wen Z, Walker SJ, et al. The MicroArray Quality Control (MAQC)-II study of common practices for the development and validation of microarray-based predictive models. *Nat Biotechnol*. 2010;28(8):827-838.
4. Mulligan G, Mitsiades C, Bryant B, Zhan F, Chng WJ, Roels S, et al. Gene expression profiling and correlation with outcome in clinical trials of the proteasome inhibitor bortezomib. *Blood*. 2007;109(8):3177-3188.
5. Chng WJ, Kumar S, Vanwier S, Ahmann G, Price-Troska T, Henderson K, et al. Molecular dissection of hyperdiploid multiple myeloma by gene expression profiling. *Cancer Res*. 2007;67(7):2982-2989.
6. Zhang Z, Hernandez K, Savage J, Li S, Miller D, Agrawal S, et al. Uniform genomic data analysis in the NCI Genomic Data Commons. *Nat Commun*. 2021;12(1):1226.
7. Barrett T, Wilhite SE, Ledoux P, Evangelista C, Kim IF, Tomashevsky M, et al. NCBI GEO: archive for functional genomics data sets--update. *Nucleic Acids Res*. 2013;41(Database issue):D991-995.
8. Zhan F, Barlogie B, Arzoumanian V, Huang Y, Williams DR, Hollmig K, et al. Gene-expression signature of benign monoclonal gammopathy evident in multiple myeloma is linked to good prognosis. *Blood*. 2007;109(4):1692-1700.
9. Richardson PG, Sonneveld P, Schuster MW, Irwin D, Stadtmauer EA, Facon T, et al. Bortezomib or high-dose dexamethasone for relapsed multiple myeloma. *N Engl J Med*. 2005;352(24):2487-2498.
10. Richardson PG, Barlogie B, Berenson J, Singhal S, Jagannath S, Irwin D, et al. A phase 2 study of bortezomib in relapsed, refractory myeloma. *N Engl J Med*. 2003;348(26):2609-2617.
11. Jagannath S, Barlogie B, Berenson J, Siegel D, Irwin D, Richardson PG, et al. A phase 2 study of two doses of bortezomib in relapsed or refractory myeloma. *Br J Haematol*. 2004;127(2):165-172.
12. Chng WJ, Huang GF, Chung TH, Ng SB, Gonzalez-Paz N, Troska-Price T, et al. Clinical and biological implications of MYC activation: a common difference between MGUS and newly diagnosed multiple myeloma. *Leukemia*. 2011;25(6):1026-1035.
13. Franzen O, Gan LM, Bjorkegren JLM. PanglaoDB: a web server for exploration of mouse and human single-cell RNA sequencing data. *Database (Oxford)*. 2019;2019.
14. Cheng C, Yan X, Sun F, Li LM. Inferring activity changes of transcription factors by binding association with sorted expression profiles. *BMC Bioinformatics*. 2007;8:452.

15. Varn FS, Andrews EH, Mullins DW, Cheng C. Integrative analysis of breast cancer reveals prognostic haematopoietic activity and patient-specific immune response profiles. *Nat Commun*. 2016;7:10248.
16. Varn FS, Wang Y, Mullins DW, Fiering S, Cheng C. Systematic Pan-Cancer Analysis Reveals Immune Cell Interactions in the Tumor Microenvironment. *Cancer Res*. 2017;77(6):1271-1282.
17. Barbie DA, Tamayo P, Boehm JS, Kim SY, Moody SE, Dunn IF, et al. Systematic RNA interference reveals that oncogenic KRAS-driven cancers require TBK1. *Nature*. 2009;462(7269):108-112.
18. Wu T, Hu E, Xu S, Chen M, Guo P, Dai Z, et al. clusterProfiler 4.0: A universal enrichment tool for interpreting omics data. *Innovation (Camb)*. 2021;2(3):100141.
19. Altboum Z, Steuerman Y, David E, Barnett-Itzhaki Z, Valadarsky L, Keren-Shaul H, et al. Digital cell quantification identifies global immune cell dynamics during influenza infection. *Mol Syst Biol*. 2014;10:720.
20. Danziger SA, Gibbs DL, Shmulevich I, McConnell M, Trotter MWB, Schmitz F, et al. ADAPTS: Automated deconvolution augmentation of profiles for tissue specific cells. *PLoS One*. 2019;14(11):e0224693.
21. Newman AM, Liu CL, Green MR, Gentles AJ, Feng W, Xu Y, et al. Robust enumeration of cell subsets from tissue expression profiles. *Nat Methods*. 2015;12(5):453-457.
22. Wilkerson MD, Hayes DN. ConsensusClusterPlus: a class discovery tool with confidence assessments and item tracking. *Bioinformatics*. 2010;26(12):1572-1573.
23. Tate JG, Bamford S, Jubb HC, Sondka Z, Beare DM, Bindal N, et al. COSMIC: the Catalogue Of Somatic Mutations In Cancer. *Nucleic Acids Res*. 2019;47(D1):D941-D947.

Supplementary Figure 1. Effects of PBM score on MM prognosis.

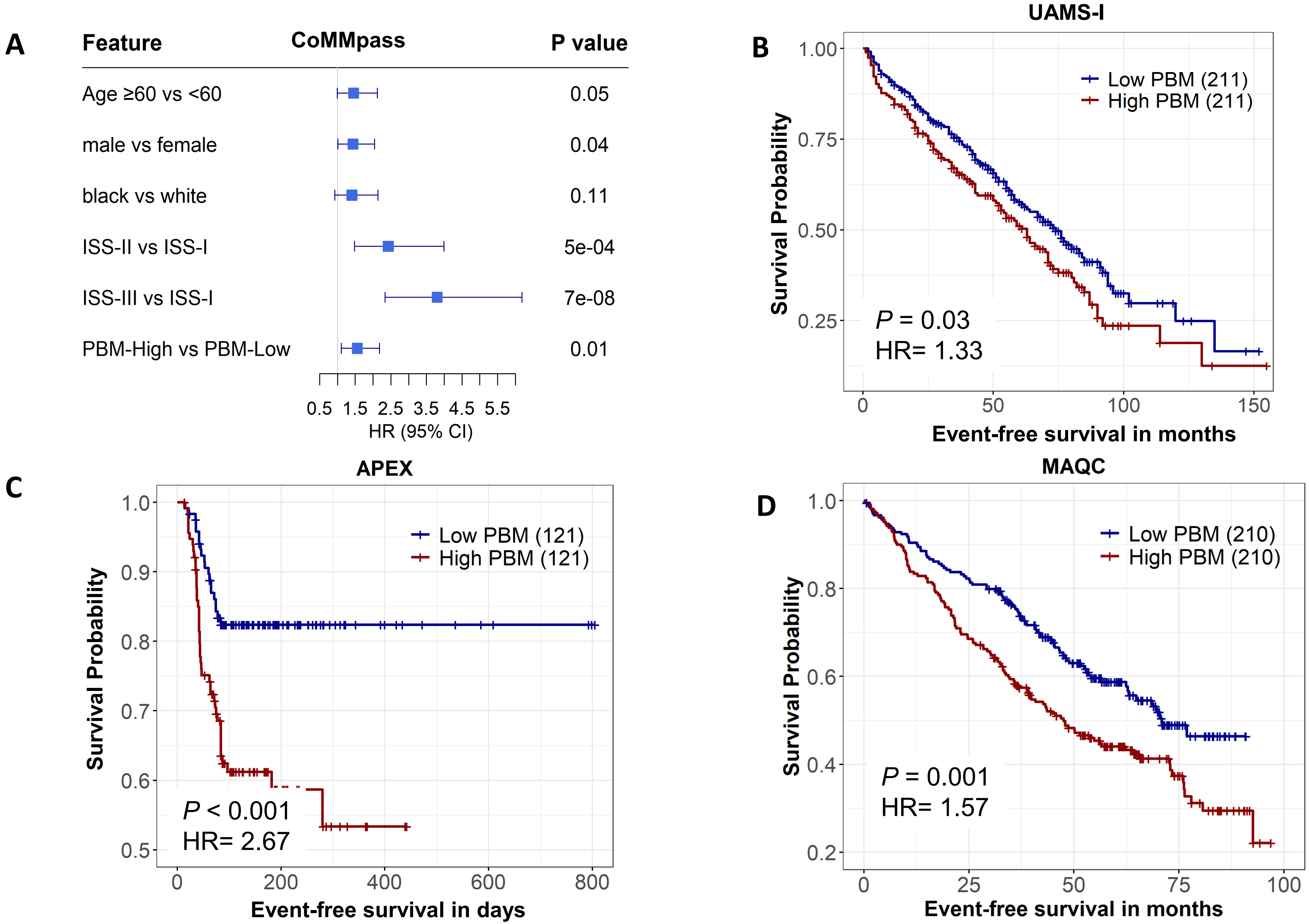

Supplementary Figure 2. PBM score predicts MM survival.

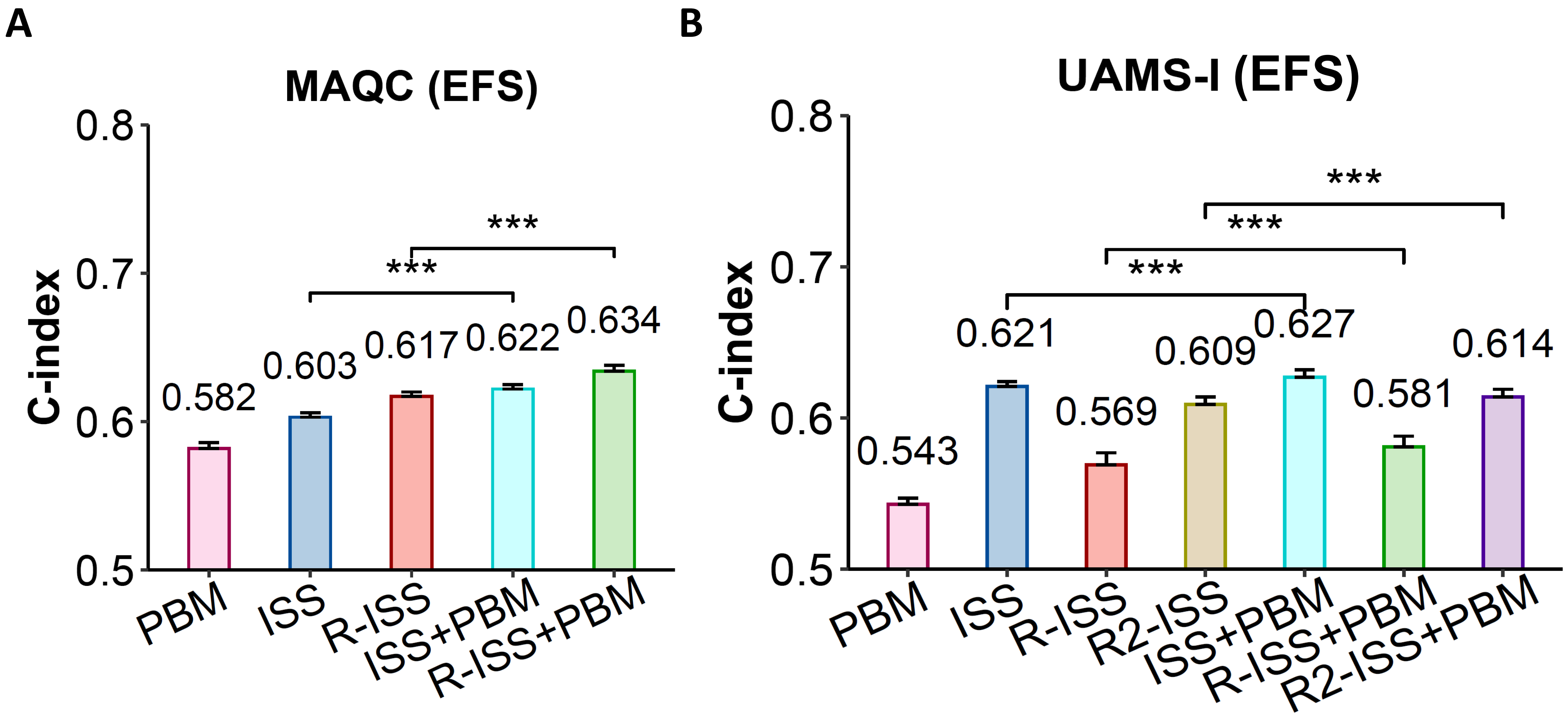

Supplementary Figure 3. Prognostic performance of the PBM score and clinical factors.

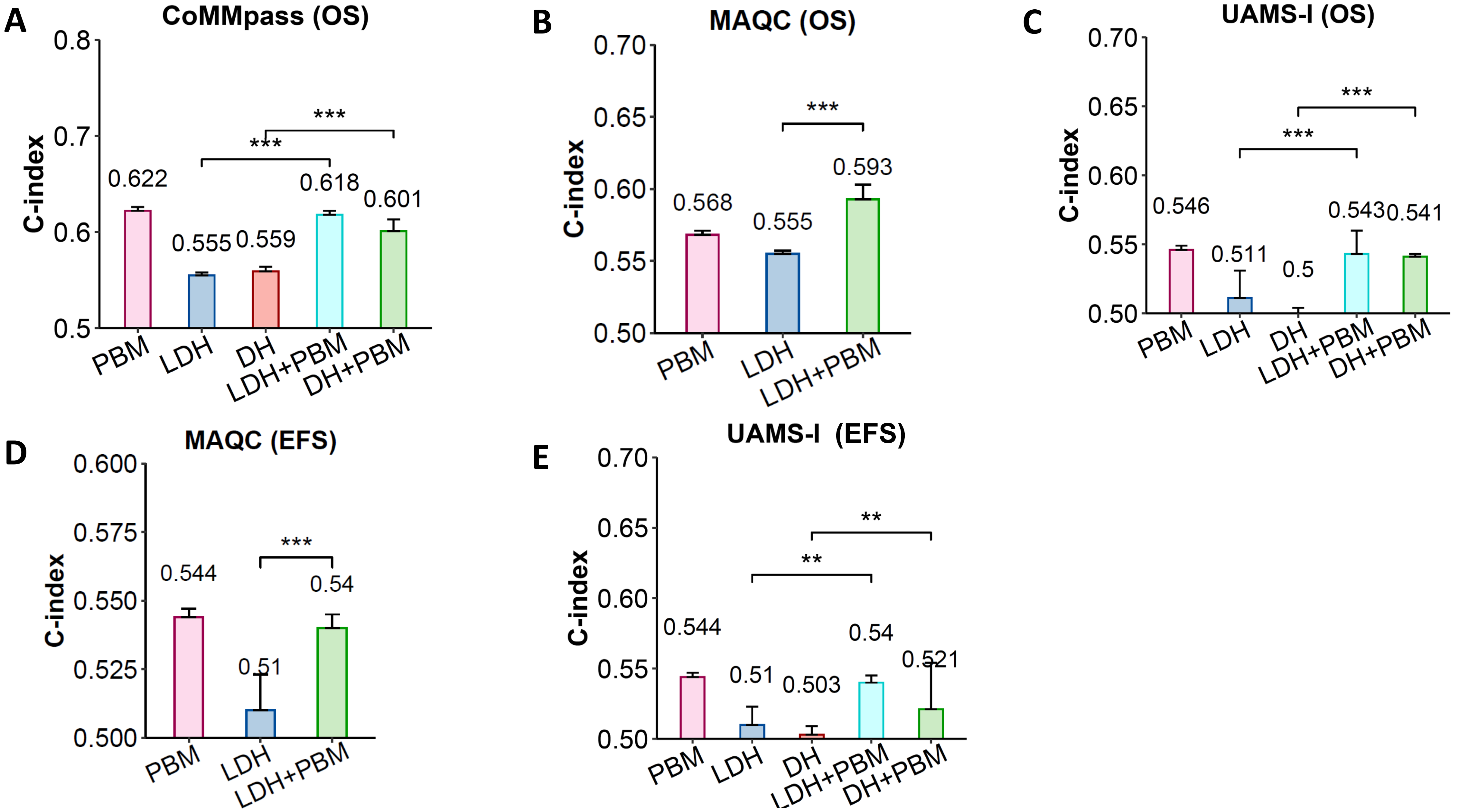

Supplement: Supplementary file 1 — Supplementary Methods and Figures [file 41408_2024_1024_MOESM1_ESM.pdf]
